# Nitrogen modulation of legume root architecture signaling pathways involves phytohormones and small regulatory molecules

**DOI:** 10.3389/fpls.2013.00385

**Published:** 2013-10-01

**Authors:** Nadiatul A. Mohd-Radzman, Michael A. Djordjevic, Nijat Imin

**Affiliations:** Division of Plant Sciences, Research School of Biology, College of Medicine, Biology and Environment, The Australian National UniversityCanberra ACT, Australia

**Keywords:** nitrogen regulation, legumes, root development, lateral root, nodulation, phytohormone, microRNA, small regulatory peptides

## Abstract

Nitrogen, particularly nitrate is an important yield determinant for crops. However, current agricultural practice with excessive fertilizer usage has detrimental effects on the environment. Therefore, legumes have been suggested as a sustainable alternative for replenishing soil nitrogen. Legumes can uniquely form nitrogen-fixing nodules through symbiotic interaction with specialized soil bacteria. Legumes possess a highly plastic root system which modulates its architecture according to the nitrogen availability in the soil. Understanding how legumes regulate root development in response to nitrogen availability is an important step to improving root architecture. The nitrogen-mediated root development pathway starts with sensing soil nitrogen level followed by subsequent signal transduction pathways involving phytohormones, microRNAs and regulatory peptides that collectively modulate the growth and shape of the root system. This review focuses on the current understanding of nitrogen-mediated legume root architecture including local and systemic regulations by different N-sources and the modulations by phytohormones and small regulatory molecules.

## INTRODUCTION

Understanding how plants grow and develop under diverse environmental conditions is crucial for improving crop productivity. As plants are sessile, they are highly sensitive to the environment and respond accordingly for growth and survival. Of particular importance is nitrogen (N) which provides the building blocks for protein production in plants and dictates crop yield and productivity. The root system adapts to soil N-levels by modifying its architecture (Hodge, 2006). In legumes, during N-limitation, specialized root organs called nodules, can form through symbiotic interaction with rhizobia which are specialized nitrogen-fixing bacteria. Rhizobia convert atmospheric N_2_ to ammonium to provide legumes with N for growth. Some of this fixed N is recycled back into the soil to sustain subsequent plant growth. Due to this ability, legumes are used as rotational or cover crops to replenish soil N (Collette et al., 2011). As legume root architecture is strongly regulated by N, understanding N-regulation of root development has great agricultural importance. The recent discovery of small regulatory molecules such as microRNAs and regulatory peptides provide additional facets to the classic phytohormone mediated pathways of root development. Therefore this review aims to give a brief perspective on the current knowledge of the signaling components involved in N-mediated root architecture with emphasis on the legume root system.

## IMPROVING PLANT ROOT ARCHITECTURE FOR BETTER N USE EFFICIENCY

Nitrogen levels strongly influence root architecture and crop yields (Hodge, 2006; Garnett et al., 2009). The enhanced crop production during the Green Revolution was mostly attributed to N fertilizer use to alleviate soil N-limitation (Tilman, 1998; Xu et al., 2012). However, there is also an internal control in plants for N use – N use efficiency (NUE) which determines the efficiency of a plant to transport, assimilate and uptake N from the environment (Xu et al., 2012). Poor NUE often translates into utilization of only 30–40% of externally supplied N and this wastage is exacerbated by the energy intensiveness of the Haber-Bosch process which consumes 1–2% of the world energy supply (Cocking, 2009; Hao et al., 2011). In addition, current food crop production has reached a NUE plateau, limiting further yield increases. Poor NUE has also led to excessive N fertilizer usage, generating adverse environmental effects including nitrate-derived water pollution, the production of reactive N, algal blooms and water and soil acidification (Tilman, 1998; Galloway et al., 2008; Rockstrom et al., 2009). Therefore, suboptimal NUE poses a major challenge. On one hand, crop production must increase to sustain world population growth (Collette et al., 2011). On the other, the further environmental damage that will ensue if NUE is not improved will undermine these efforts. Disturbances in the global N-cycle are already negatively impacted global biosphere health and reactive N gases contribute to global warming (Rockstrom et al., 2009). A more sustainable alternative involves utilizing the biological N fixation ability of legumes to replenish soil N. Therefore, a better understanding of legume development with respect to N-mediated root growth is required for agricultural sustainability.

## IMPORTANCE OF LEGUMES TO PROVIDE AN ALTERNATIVE N-SOURCE IN SUSTAINABLE AGRICULTURE

Nitrogen-fixing root nodules form from the legume-*Rhizobium* symbiosis. A “zone of maximum susceptibility” occurs in the elongation zone near the root tip (Bhuvaneswari et al., 1980; Sargent et al., 1987). *Rhizobium*-derived nodulation (Nod) factors are required to induce root hair curling, infection and nodule primordium formation. Rhizobia colonize mature nodules and fix N. The legume-*Rhizobium* symbiosis contributes between 14 and 140 kg of N/acre/year and 33% of human protein globally (Graham and Vance, 2003). A 15 year study involving the co-cultivation of maize with soybean compared to growing maize grown alone showed a significant reduction of carbon and N loss to the environment (Drinkwater et al., 1998). The Food and Agriculture Organization (FAO) promotes sustainable agriculture by increasing legume usage in crop-rotations and as cover crops to enrich soil N levels (FAO, 2009; Collette et al., 2011). Although N-limitation has been long known as a prerequisite for nodulation to occur, the mechanism behind legume root susceptibility for nodulation is still unknown. Since lateral root and nodule development and overall root architecture are strongly influenced by N-availability, a comprehensive understanding of these processes is required to optimize legume utilisation for sustainable agriculture.

## N REGULATION OF ROOT ARCHITECTURE IS MEDIATED THROUGH SYSTEMIC AND LOCAL SIGNALING PATHWAYS

**FIGURE 1:**
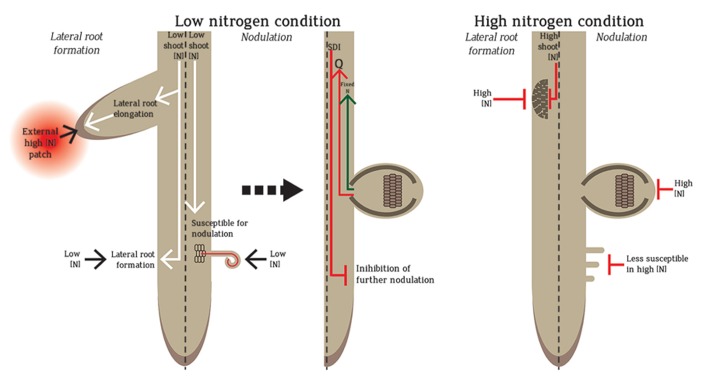
**Regulation of nodule and lateral root formation in low and high N.** Low N promotes the formation of lateral roots and nodules. Lateral root formation increases in low N to promote foraging. However, if the root senses a patch of high N during N-limitation, lateral root elongation is promoted towards the high N-patch to exploit the available N-source for the plant use. In low N, legume roots are susceptible to rhizobial infection and form nodules in which N is fixed by rhizobia and transported into the plant. The formation of nodules also sends a root-derived signal “Q” to the shoot, which triggers the production of a shoot-derived inhibitor (SDI) that travels back to the root to inhibit further nodulation. In homogenous high N conditions, both lateral roots and nodules are inhibited. In high N, the root is less susceptible for nodulation and infection, nodule number development and N fixation capacity are reduced. High shoot N also leads to less lateral root formation.

Local and systemic controls influence N-mediated root architecture regulation (**Figure 1**). In addition, homogenous and heterogeneous N-regimes impart differential responses in dicots and monocots. Local control is exemplified by the stimulation of lateral root elongation by high N-patches in the soil (Robinson et al., 1999). In the systemic pathway, root architecture is dictated by the plant’s overall N-status (Zhang and Forde, 1998; Robinson et al., 1999). Homogeneous high nitrate (e.g. ≥10 mM) imparts systemic inhibition of lateral and primary root growth (**Figure 1**) whereas homogeneous low nitrate (e.g. ≤1 mM) promotes both (Robinson et al., 1999; Zhang et al., 1999; Walch-Liu et al., 2006; Ruffel et al., 2011). Coordinated systemic and local regulations are observed in split-root experiments where the root system is split into two, with each side exposed to different treatments. Split-root exposed to low and high N-level on each side respectively shows more lateral roots form on the side exposed to high nitrate (Ruffel et al., 2011). The root foraging mechanism exploits the high N-patches and minimal investment is made by the plant to the N-limited roots (Robinson et al., 1999). Compared to these N-regulations of lateral roots, less is known about nodule regulation by local and systemic N pathways.

Local and systemic pathways also regulate nodule numbers. The earliest formed nodules stimulate systemic autoregulation which suppresses further nodulation in younger root regions (**Figure 1**). Autoregulation can also be observed in split-root experiments: nodules forming on one split-root will inhibit nodulation on the second split-root (Kosslak and Bohlool, 1984; Sargent et al., 1987). Nodules formed by the first inoculation produce root-derived signal (Q) which travels to the shoot via the xylem to be ultimately perceived by a leucine-rich repeat receptor-like kinase (LRR-RLK). Functional orthologues of this LRR-RLK have been identified in *Lotus japonicus* (HAR1; hypernodulation aberrant root 1), *Medicago truncatula* (SUNN; super numeric nodules), soybean (NARK; nodule autoregulation receptor kinase) and pea (SYM29; Krusell et al., 2002; Searle et al., 2003; Schnabel et al., 2005). After Q perception, the shoots produce a shoot-derived inhibitor (SDI) that suppresses nodulation in other parts of the roots (**Figure 1**; Caetano-Anolles and Gresshoff, 1991). Systemic control is also observed through selective discrimination of rhizobia based on N-fixation and nodulation efficiency (Sargent et al., 1987; Laguerre et al., 2012). Local high nitrate strongly inhibits nodulation locally and initiates a systemic response. The systemic response can be measured using split-roots (Ruffel et al., 2008; Jeudy et al., 2010). As plants need to invest a lot of energy to maintain nodules, the coordinate regulation of local and systemic pathways ensures that plants have sufficient N with the least energy investment. Therefore, plants will opt for nodulation only when they have a high requirement for N but utilize an external N source whenever available.

## INORGANIC AND ORGANIC N INFLUENCE ON PLANT ROOT DEVELOPMENT

Plants take up N from the soil in either inorganic (e.g., nitrate or ammonium) or organic (e.g., amino acids) forms. Most plants prefer nitrate to ammonium because excess nitrate can be stored in vacuoles and high ammonium can be toxic (Glass et al., 2002). In legumes, high nitrate and ammonium (>3 mM) inhibit nodulation but lower concentrations (0.5–2 mM) can stimulate nodulation (Bollman and Vessey, 2006; Barbulova et al., 2007). Different N-sources regulate roots differentially (Bollman and Vessey, 2006; Ruffel et al., 2008). Split-root experiments comparing the effects of growth by nitrate, ammonium or N-fixation revealed that nitrate is the only N-source that compensates growth during N-limitation (Ruffel et al., 2008; Jeudy et al., 2010). When one side of the split-root is in sufficient nitrate and the other is N-limited, the root will compensate the systemic N-limitation by increasing nitrate uptake from the sufficient side (Jeudy et al., 2010). Both ammonium and N-fixation do not seem to have compensatory regulation for growth during N-limitation. However, long-term N-limitation leads to nodule growth stimulation in the sufficient N-side of the split-root and inhibits nodulation in the N-limited side (Salon et al., 2009; Jeudy et al., 2010). These differential root responses to nitrate, ammonium and N-fixation demonstrate the ability of legumes to distinguish between the N-regimes. This is likely to be mediated by different sensory components regulating the plant responses to the respective N-forms.

Root growth in ammonium is partly regulated by ammonium transporters (AMTs) which are involved in maintaining optimal ammonium levels *in planta* and modulating root responses to prevent ammonium toxicity. The AMT1 and AMT2 families were identified in *Arabidopsis* (Yuan et al., 2007). The AMT1 family controls ammonium transport and acquisition while the AMT2 family is involved in regulatory processes (Sohlenkamp et al., 2002; Yuan et al., 2007). In *Lotus*, three AMT1s and two AMT2s have been characterized (Salvemini et al., 2001; Simon-Rosin et al., 2003; D’Apuzzo et al., 2004; Rogato et al., 2010a). LjAMT1;1 and LjAMT1;2 are up-regulated during N-limitation. LjAMT1;3 is up-regulated by high ammonium (D’Apuzzo et al., 2004) and is a putative ammonium transceptor that mediates root responses to toxic ammonium levels (Rogato et al., 2010a, b). LjAMT2;1 is postulated to recover ammonium lost from cellular efflux in nodules and other organs (Simon-Rosin et al., 2003) whereas LjAMT2;2 is required for N-acquisition during mycorrhizal associations (Guether et al., 2009). Ammonium negatively impacts nodulation by inhibiting root hair curling and repressing the expression of *NIN* (*NODULE INCEPTION*), an essential gene for nodule formation (Barbulova et al., 2007). These results indicate that ammonium inhibition is upstream of the Nod factor pathway and that ammonium perception needs to be relayed quickly for rapid nodule inhibition to occur.

Nitrate, the predominant form of soil inorganic N, strongly affects lateral root and nodule formation. In contrast to ammonium, nitrate inhibition occurs downstream of the nodulation pathway just before cortical cell division (Barbulova et al., 2007). The *Lotus* autoregulation mutant, *har1,* is nitrate-insensitive but retains sensitivity to ammonium. Autoregulation mutants from other species are also nitrate insensitive suggesting that nitrate is involved in the autoregulation pathway (Schnabel et al., 2010). Two nitrate transporter families, NRT1 and NRT2, mediate nitrate-dependent responses. NRT1s are mostly low affinity transporters (LATs) and NRT2 are mostly high affinity transporters (HATs; Tsay et al., 2007). In *Medicago*, two NRT1 transporters were identified: NIP/LATD (numerous infections and polyphenolics/lateral root-organ defective), which is involved in root architecture regulation (Bagchi et al., 2012), and NRT1.3, which regulates nitrate uptake in N-deficient conditions (Morère-Le Paven et al., 2011). NIP/LATD acts as a HAT under low nitrate conditions and NIP/LATD mutants have severe defects during nodule and lateral root formation, which can only be partially rescued by an *Arabidopsis *NRT1 homologue (Bagchi et al., 2012). This suggests additional functions for NIP/LATD apart from transporting nitrate (Yendrek et al., 2010; Bagchi et al., 2012). MtNRT1.3, which encodes a dual-affinity nitrate transporter similar to AtNRT1.1, is postulated to regulate nitrate uptake during N-limitation (Morère-Le Paven et al., 2011). Since AtNRT1.1 is known to be a transceptor, the legume NRT homologs could be involved in nitrate-dependent nodulation signaling pathways. Nitrate inhibits nodulation not because of its nutritional effect but more likely as an important signaling cue to regulate nodulation (Carroll and Mathews, 1990).

Apart from inorganic N, free amino acids, particularly glutamine, also affect root architecture. High glutamine inhibits root growth by acting as an internal N-status signal for mediating root development (Zhang et al., 1999). In legumes, the glutamine, asparagine and ureides produced by nodules may also regulate nodulation. A high level of fixed-N in the phloem lowers nitrogenase activity in nodules (Parsons et al., 1993; Imsande and Touraine, 1994; Sulieman et al., 2010). These reductions in nitrogenase activity through feedback regulation might contribute to nodule modulation (Parsons et al., 1993; Serraj and Sinclair, 2003; Sulieman and Tran, 2013).

N-mediated regulation of root development ensures that sufficient N acquisition occurs to support the formation of the photosynthetic apparatus in the shoot. The shoot then invests carbon to promote root development to explore the soil for more N or initiate symbiosis to fix N (Garnett et al., 2009; Nunes-Nesi et al., 2010). Therefore, the communication between local and systemic pathways is tightly regulated by phytohormones and other regulatory molecules including small regulatory molecules.

## PHYTOHORMONES: WELL-KNOWN MEDIATORS OF LONG-RANGE SIGNALING IN N-REGULATION

Auxin is the major hormone implicated in root development and N-mediated control of root architecture. High nitrate is thought to reduce local auxin accumulation suggesting that auxin may be a shoot-to-root signal of “N-status” (Bao et al., 2007; Okushima et al., 2011). In *Arabidopsis*, high shoot N-levels are speculated to reduce shoot-to-root auxin transport resulting in reduced lateral root formation (Reed et al., 1998; Forde, 2002). However studies with *Medicago* reveals that high shoot N-levels increase shoot-to-root auxin transport (Jin et al., 2012). The correlation between shoot-to-root auxin transport and lateral root formation suggests that systemic N-regulation via auxin acts through modulating auxin levels for the formation of lateral root founder cells (Dubrovsky et al., 2011). This response is demonstrated by using *Medicago* *sunn-1* mutant, which has insensitive shoot-to-root auxin transport regardless of the N-level. In *sunn-1*, the correlation between N-dependent auxin transport and lateral root regulation is lost (Jin et al., 2012). However, SUNN-dependent shoot-to-root auxin transport seems to only apply to nitrate-mediated lateral root regulation but not nodule regulation suggesting that auxin-dependent N-regulation of nodulation acts locally in the root (Jin et al., 2012). Auxin regulation of root development involves crosstalk with other phytohormones such as ethylene.

The gaseous hormone, ethylene, is directly regulated by soil nitrate levels and is involved in local nitrate-dependent root regulation. In several species, high nitrate increases root ethylene evolution (Ligero et al., 1986, 1987; Caba et al., 1998; Tian et al., 2009). High ethylene levels inhibit nodules and lateral roots formation while low ethylene increases lateral roots and promotes nodulation (Peters and Crist-Estes, 1989; Lee and Larue, 1992; Nukui et al., 2000; Oldroyd et al., 2001). Several rhizobia generate better nodulation responses by inhibiting localized ethylene by synthesizing an ethylene precursor mimic or by producing the aminocyclopropane-deaminase enzyme which degrades aminocyclopropane, the ethylene precursor (Ma et al., 2002). Ethylene also imparts positional control of nodulation as increased ethylene levels opposite the phloem poles favors nodule formation opposite the xylem poles (Heidstra et al., 1997). Ethylene regulation of lateral roots and nodules likely occurs through the control of cell cycle pathways (Dan et al., 2003; Spadafora et al., 2012) however little detail is known how this occurs. Cell cycle regulation by ethylene occurs partly through crosstalk with cytokinin (Spadafora et al., 2012) which is also involved in nitrate-regulated development.

Cytokinin directly regulates the cell cycle and is a mediator for communicating N-status between the shoot and root via the phosphorelay pathway (Sakakibara et al., 2000). In this pathway, nitrate replenishment of an N-starved root system increases cytokinin synthesis which is then transported to the shoot, signaling the root’s N status. As shoot N-supply is depleted, cytokinin is transported back to the root to signal the shoot’s low N-status (Sakakibara et al., 2000; Ruffel et al., 2011). Cytokinin also regulates the cell cycle during lateral root development by acting directly on lateral root founder cells to inhibit root initiation (Li et al., 2006; Laplaze et al., 2007). However, once differentiation occurs, high cytokinin promotes lateral root elongation (Li et al., 2006). As lateral root elongation is also stimulated by high nitrate patches, it would be interesting to examine the cytokinin-nitrate interaction during this process. In legumes, cytokinin is an upstream component of the nodulation pathway and exogenous cytokinin application induces the expression of several nodulation genes (Fang and Hirsch, 1998; Gonzalez-Rizzo et al., 2006). Cytokinin receptor mutants of *Lotus* (Murray et al., 2007) and *Medicago* (Gonzalez-Rizzo et al., 2006) show reduced nodulation while a cytokinin receptor gain-of-function mutants leads to spontaneous nodulation (Murray et al., 2007; Tirichine et al., 2007). Although N-mediated root development involves cytokinin, auxin and ethylene, small regulatory molecules fine-tune these pathways.

## SMALL REGULATORY MOLECULES FOR FINE-TUNING PLANT DEVELOPMENTAL RESPONSES

Regulatory microRNAs and peptides act as fine-tuners of local cellular development. These small regulatory molecules are likely to act as cellular cues in response to environmental conditions including N-availability. The signaling cascades then activate the phytohormone pathways to modulate the root system. For example, the auxin receptor, Auxin signaling F-box protein 3 (AFB3), is a target of microRNA, miR393 (Vidal et al., 2010). Since AFB3 and miR393 are both nitrate-induced, this leads to a transient up-regulation of AFB3 in a feed-forward loop prior to the subsequent induction of miR393 which down-regulates AFB3 (Vidal et al., 2010). The rapid down-regulation of AFB3 by miR393 provides a fine-tuned mechanism of the root system to dynamically respond to N in real time. This interaction is an excellent example of a small regulatory molecule integrating nitrate availability with auxin signaling. Recently, soybean miR160 has also been shown to modulate auxin during nodulation. miR160 is down-regulated by *Rhizobium* inoculation while its *Arabidopsis* homologue is upregulated by N-starvation (Subramanian et al., 2008; Liang et al., 2012). Over-expressing miR160 in soybean leads to auxin hypersensitivity which reduces nodule formation demonstrating the importance of auxin regulation by miR160 during nodulation (Turner et al., 2013). miR169 is also involved in N-regulation of root development. Overexpressing miR169 in *Arabidopsis* reduces nuclear factor Y-A (NF-YA) transcript levels leading to a low N-stress phenotype coupled with low N acquisition (Zhao et al., 2011). In *Medicago*, miR169 targets HAP2-1, a NF-YA involved in nodulation (Combier et al., 2006). This *Medicago* NF-YA is closely regulated with *NIN* (Soyano et al., 2013). *NIN* and its *Arabidopsis* homolog, *NLP7* are up-regulated by low N (Wang et al., 2009). As *nlp7* displays N-stressed root phenotypes even in N-sufficient conditions (Castaings et al., 2009), it would be interesting to see if miR169 or other regulatory molecules are involved in NIN/NLP7 dependent pathway during N-limitation in roots. 

Other regulatory molecules in plants include small signaling peptides. The most well-studied regulatory peptide families in plants are the CLE peptide family. Several members of this family are involved in root development including CLE40, which involves in the maintenance of the root apical meristem (RAM) by regulating cellular differentiation (Stahl et al., 2009). In legumes, nodule-specific CLE peptides also regulate nodulation (Saur et al., 2011; Mortier et al., 2012). Several CLEs are nitrate-regulated like GmNIC1 in soybean which is involved in the autoregulation of nodulation (Reid et al., 2011, 2013). During autoregulation, GmNIC1 interacts with the NARK receptor in the root (Reid et al., 2011, 2013). Recently, the LjCLE-RS2 peptide in *Lotus* was demonstrated to be the “Q” signal (**Figure 1**) which interacts with HAR1 in the shoot (Okamoto et al., 2013). LjCLE-RS2 is also up-regulated by nitrate and is hypothesized to integrate nitrate inhibition of nodulation via the HAR1-dependent autoregulation pathway (Okamoto et al., 2009). Nodule inhibition by these nitrate-regulated CLEs suggests possible crosstalks between autoregulation and nitrate-regulation of nodulation. These CLEs are likely to be involved in signal transduction pathways which further regulate the cytokinin-mediated nodulation pathway (Saur et al., 2011). Therefore, these small regulatory peptides provide a fine-tuning mechanism for nitrate-mediated control of root architecture.

## CONCLUSION

Nitrogen is an essential nutrient for plant productivity and its environmental availability strongly regulates root architecture. To optimize N acquisition, nitrate and ammonium transporters/transceptors provide the sensory components of N-mediated root development in legumes. The signal for N-availability is translated into an array of phytohormone pathways which regulates root development. Small regulatory molecules such as microRNAs and peptides provide further fine-tuning of these phytohormone signals to produce highly dynamic and plastic root responses to N-levels. Hence these regulatory pathways, which integrate environmental sensory signals with the modulation of phytohormones and small regulatory molecules could be exploited to improve legume root architecture for better NUE.

## Conflict of Interest Statement

The authors declare that the research was conducted in the absence of any commercial or financial relationships that could be construed as a potential conflict of interest.

## Author Contributions

Nadiatul A. Mohd-Radzman wrote the paper. Nijat Imin and Michael A. Djordjevic edited the paper.
